# Supplementary material for: M-Carboxylic Acid Induced Formation of New Coordination Polymers for Efficient Photocatalytic Degradation of Ciprofloxacin
Source: Molecules. 2022 Nov 10;27(22):7731. doi: 10.3390/molecules27227731 (PMC9693974; doi:10.3390/molecules27227731)
Supplement: Supplementary file 1 [file molecules-27-07731-s001.zip › molecules-1936718-SI.pdf]

Supporting Information (SI)

M-Carboxylic Acid Induced Formation of New Coordination Polymers for Efficient Photocatalytic Degradation of Ciprofloxacin

Jian Li[1,2], Xiaojia Wang[1], Yunyin Niu[1,*]

1. Synthesis of the ligand L1

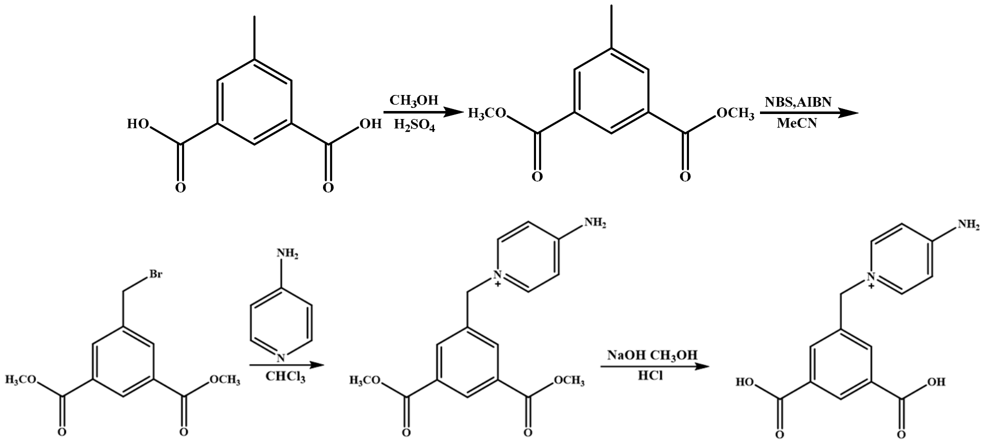

Scheme S1. Synthesis route of the cationic template L1.

2.

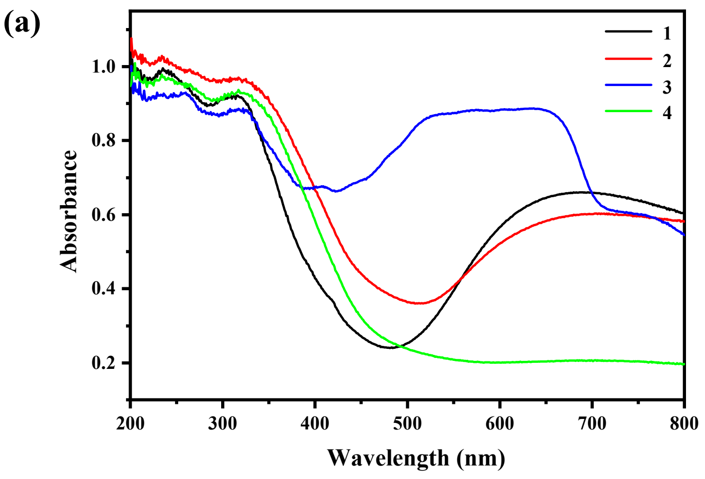

FigureS1. The diffuse reflectance UV-Vis spectra of Compounds **1-4**.

The band gap energy (Eg) of compounds **1-4** was calculated according to $Ahv = C(hv-Eg)^2$, where A is the absorption coefffcient, h is Planck constant, v is incident light frequency, and C is the constant. [Materials Chemistry and Physics **256** (2020) 123650]

3.Synthesis of **2**

Compound **2** was synthesized in a procedure analogous to that of **1** except that the pH of the solution was adjusted to 3. The resulting product was recovered by filtration, washed with distilled water, and dried in air. Yield: 16%. IR (KBr, cm^{-1}): 3419.83 (s), 2923.50 (m), 2853.07 (s), 1649.42 (s), 1616.20 (m), 1573.44 (w), 1285.10 (m), 1222.53 (m), 1173.54 (w), 1071.87 (w), 944.90 (m), 856.44 (w), 741.54 (w). Elemental Anal. Calc. for $C_{38}H_{30}Cu_2Mo_5N_6O_{24}$ (1561.46): C, 29.23; H, 1.93; N, 5.38. Found: C, 29.27; H, 1.89; N, 5.41.

4. Synthesis of **3**

A solution of L1 (0.0093 g，0.03 mmol)，$CoCl_2·6H_2O$ (0.0072 g,0.03 mmol), H_2O (1mL) and DMA (3 mL) was stirred under ambient conditions, and adjust the pH to 3 with HCl (2 M), then sealed in a Teflonlined steel autoclave, heated at 120 °C for 3 days, and cooled to room temperature. The resulting product was recovered by filtration, washed with distilled water, and dried in air. Yield: 35%. IR (KBr, cm^{-1}): 3419.53 (s), 2923.20 (w), 1655.83 (s), 1614.86 (m), 1571.01 (m), 1540.87 (m), 1454.83 (w), 1411.81 (m), 1362.81 (m), 1035.46 (w), 936.04 (w), 775.93 (m), 627.23 (w). Elemental Anal. Calc. for $C_{28}H_{28}CoN_4O_{11}$ (655.20): C, 51.32; H, 4.27； N, 8.55. Found: C, 51.29; H, 4.23; N, 8.51.

5. Synthesis of **4**

A solution of L1 (0.0086 g, 0.025 mmol)，$(NH_4)_6Mo_7O_{24}·4H_2O$ (0.0309 g，0.025 mmol) and H_2O (10 mL) was stirred under ambient conditions, and adjust the pH to 3 with HCl(2 M), then sealed in a Teflonlined steel autoclave, heated at 120 °C for 3 days, and cooled to room temperature. The resulting product was recovered by filtration, washed with distilled water, and dried in air. Yield: 32%. IR (KBr, cm^{-1}): 3382.34 (s), 3204.34 (m), 1715.70 (m), 1648.94 (s), 1562.73 (w), 1540.34 (m), 1511.74 (w), 1453.49 (w), 1172.22 (m), 942.46 (m), 913.84 (s), 713.72 (m), 666.16 (m). Elemental Anal. Calc. for $C_{28}H_{36}Mo_4N_4O_{26}$ (1228.05): C, 27.28; H, 2.98; N, 4.51. Found: C, 27.31; H, 3.01; N, 4.47.

Table S1 Crystal data and structure refinement details for **1-4**

**compounds**	**1**	**2**	**3**	**4**
Empirical formula	$C_{19}H_{23}CuMo_4N_3O_{20}$	$C_{19}H_{15}CuMo_{2.5}N_3O_{12}$	$C_{28}H_{28}CoN_4O_{11}$	$C_{28}H_{36}Mo_4N_4O_{26}$
Formula weight	1060.70	780.73	655.47	1228.37
Temperature/K	296.15	296.15	273(2)	296.15
Crystal system	triclinic	monoclinic	orthorhombic	triclinic
Space group	P-1	I2/c	Pbcn	P-1
a/Å	10.1807(8)	19.270(5)	12.1978(12)	9.6095(9)
b/Å	11.6937(9)	9.701(3)	16.4599(17)	13.2283(13)
c/Å	13.2145(10)	26.084(6)	14.0994(14)	15.8941(16)
α/°	81.3260(10)	90	90	91.628(2)
β/°	80.5730(10)	109.01(2)	90	90.650(2)
γ/°	68.6430(10)	90	90	100.449(2)
Volume/$Å^3$	1438.24(19)	4610(2)	2830.8(5)	1985.8(3)
Z	2	8	4	2
ρ/g cm^3	2.449	2.250	1.538	2.054
μ/mm^{-1}	2.520	2.318	0.676	1.336
F (000)	1030.0	3040.0	1356.0	1216.0
Crystal size/mm^3	0.22×0.05×0.06	0.12×0.11×0.1	0.26×0.16×0.15	0.12×0.3×0.06
Reflections collected	8658	10861	13594	12121
Independent reflections	6288 [R_{int}= 0.0165, R_{sigma}= 0.0385]	4050 [R_{int}= 0.1160, R_{sigma}= 0.1414]	2497 [R_{int}0.0509, R_{sigma}= 0.0358]	8698 [R_{int}=0.0221, R_{sigma}= 0.0544]
Data/restraints /parameters	6288/0/428	4050/756/339	2497/0/201	8698/0/564
Goodness-of-fit on F^2	1.024	1.094	1.290	1.020
Final R indexes [I>=2σ (I)]	R_1= 0.0303, wR_2= 0.0634	R1= 0.1041, wR2= 0.2452	R_1= 0.0940, wR_2= 0.2602	R_1= 0.0389, wR_2= 0.0807
Final R indexes [all data]	R_1= 0.0497, wR_2= 0.0707	R_1= 0.1537, wR_2= 0.2720	R_1= 0.1053, wR_2= 0.2643	R_1= 0.0706, wR_2= 0.0945
Largest diff. peak/hole / e $Å^{-3}$	0.69/-0.77	3.88/-1.95	0.42/-0.51	0.69/-0.89

Table S2 Bond length (Å) and bond angle data of compounds **1-4**(°)

Compound **1**

Mo1-Mo2	2.344(2)	Mo4-O3	2.472(3)	Mo3-O201	2.407(3)
Mo1-Mo3	2.146(9)	Mo4-O4	1.968(3)	Mo3-O2	1.991(3)
Mo1-O20	1.484(10)	Mo4-O6	1.908(3)	Mo3-O3	2.337(2)
Mo1-O2	1.489(9)	Mo4-O71	2.280(3)	Mo3-O6	1.896(3)
Mo1-O3	1.405(15)	Mo4-O17	1.705(3)	Mo3-O8	1.689(3)
Mo1-O31	115.23(5)	Mo4-O1	1.683(3)	Mo3-O15	1.698(3)
Mo1-O5	98.28(17)	Cu1-O9	2.018(3)	O18-Cu1-O12	92.23(12)
Mo1-O7	108.6(6)	Cu1-O11	1.912(3)	N2-Cu1-O9	89.43(13)
Mo2-O20	105.4(8)	Cu1-O12	2.324(3)	N2-Cu1-O12	92.47(12)
Mo2-O21	109.5(9)	Cu1-O18	2.008(3)	N2-Cu1-O18	88.09(14)
Mo2-O3	120.4(5)	Cu1-N2	1.972(3)	Mo1-O20-Mo2	108.00(13)
Mo2-O4	112.3(9)	O16-Mo2-O20	98.22(13)	Mo1-O20-Mo31	110.97(12)
Mo2-O12	1.705(3)	O16-Mo2-O21	163.99(12)	Mo2-O20-Mo31	103.84(11)
Mo2-O16	1.690(3)	O16-Mo2-O3	93.14(12)	Mo1-O2-Mo21	109.96(12)
Mo2-Mo1-Mo3	91.614(13)	O16-Mo2-O4	101.74(14)	Mo1-O2-Mo3	108.66(12)
O20-Mo1-Mo2	36.37(8)	O16-Mo2-O12	104.55(14)	Mo3-O2-Mo21	105.06(11)
O20-Mo1-Mo3	125.57(8)	O201-Mo3-Mo1	78.71(6)	Mo1-O3-Mo11	104.32(11)
O20-Mo1-O3	78.64(10)	O2-Mo3-Mo1	35.19(7)	Mo1-O3-Mo2	91.98(9)
O20-Mo1-O31	77.16(10)	O2-Mo3-O201	70.95(10)	Mo1-O3-Mo3	91.22(9)
O2-Mo1-Mo2	125.01(8)	O2-Mo3-O3	73.27(10)	Mo11-O3-Mo4	91.09(8)
O2-Mo1-Mo3	36.15(8)	O3-Mo3-Mo1	41.85(6)	Mo1-O3-Mo4	164.54(13)
O2-Mo1-O20	149.66(11)	O3-Mo3-O201	70.76(9)	Mo2-O3-Mo11	97.49(9)
O2-Mo1-O31	77.67(10)	O6-Mo3-Mo1	119.30(8)	Mo2-O3-Mo3	162.20(13)
O2-Mo1-O3	78.95(10)	O6-Mo3-O201	81.83(11)	Mo2-O3-Mo4	87.02(8)
O31-Mo1-Mo2	85.90(6)	O6-Mo3-O2	145.10(11)	Mo3-O3-Mo11	98.67(9)
O3-Mo1-Mo2	46.08(6)	O6-Mo3-O3	77.46(10)	Mo3-O3-Mo4	85.25(8)
O3-Mo1-Mo3	46.93(7)	O8-Mo3-Mo1	85.62(11)	Mo2-O4-Mo4	115.94(13)
O31-Mo1-Mo3	86.85(6)	O8-Mo3-O201	163.93(12)	Mo3-O6-Mo4	117.80(14)
O3-Mo1-O31	75.68(11)	O8-Mo3-O2	98.09(13)	Mo1-O7-Mo41	119.55(13)
O3-Mo1-Mo3	46.93(7)	O8-Mo3-O3	95.14(12)	C14-O11-Cu1	127.6(3)
O31-Mo1-Mo3	86.85(6)	O8-Mo3-O6	103.09(14)	Mo2-O12-Cu1	174.58(17)
O3-Mo1-O31	75.68(11)	O8-Mo3-O15	105.48(15)	O11-Cu1-O18	93.47(13)
O5-Mo1-Mo2	91.00(10)	O15-Mo3-Mo1	135.48(10)	O11-Cu1-N2	175.55(14)
O5-Mo1-Mo3	90.45(10)	O15-Mo3-O201	88.39(12)	O18-Cu1-O9	165.88(12)
O5-Mo1-O20	101.92(13)	O15-Mo3-O2	100.32(13)	C18-N2-Cu1	120.1(3)
O5-Mo1-O2	101.94(13)	O15-Mo3-O3	159.15(13)	O11-Cu1-O12	83.31(11)
O5-Mo1-O3	100.16(12)	O15-Mo3-O6	100.32(13)	C19-N2-Cu1	122.0(3)
O5-Mo1-O31	175.82(12)	O4-Mo4-O3	73.02(10)	O12-Mo2-O20	98.91(13)
O5-Mo1-O7	104.38(14)	O4-Mo4-O71	76.64(11)	O12-Mo2-O21	89.61(12)
O7-Mo1-Mo2	131.54(9)	O6-Mo4-O3	73.87(10)	O12-Mo2-O3	161.83(12)
O7-Mo1-Mo3	132.91(9)	O6-Mo4-O4	143.91(12)	O12-Mo2-O4	101.75(13)
O7-Mo1-O20	95.18(12)	O6-Mo4-O71	78.74(11)	O16-Mo2-Mo1	84.93(10)

O7-Mo1-O2	96.76(12)	O71-Mo4-O3	69.57(9)	O4-Mo2-O20	146.48(11)
O7-Mo1-O3	155.45(11)	O17-Mo4-O3	159.06(13)	O4-Mo2-O21	82.23(11)
O7-Mo1-O31	79.79(11)	O17-Mo4-O4	101.78(13)	O4-Mo2-O3	78.21(10)
O20-Mo2-Mo1	35.63(7)	O17-Mo4-O6	103.99(13)	O12-Mo2-Mo1	134.40(10)
O20-Mo2-O21	71.77(10)	O17-Mo4-O71	89.52(13)	O3-Mo2-O21	72.32(9)
O20-Mo2-O3	74.04(10)	O1-Mo4-O3	95.52(12)	O4-Mo2-Mo1	120.15(8)
O21-Mo2-Mo1	79.74(6)	O1-Mo4-O4	96.82(13)	O9-Cu1-O12	101.76(11)
O3-Mo2-Mo1	41.94(6)	O1-Mo4-O6	100.25(14)	O11-Cu1-O9	90.05(12)
O21-Mo2-Mo1	79.74(6)	O1-Mo4-O71	164.84(13)		
O3-Mo2-Mo1	41.94(6)	O1-Mo4-O17	105.30(16)		
		Compound **2**			
Mo1-O3^{1}	2.268(12)	Mo2-Mo3	3.201(2)	Mo3-O9	1.849(11)
Mo1-O3^{2}	2.268(12)	Mo2-O1	2.282(12)	Mo3-O10	2.082(12)
Mo1-O5	1.690(12)	Mo2-O6	1.963(11)	Mo3-O10^{4}	2.046(12)
Mo1-O5^{3}	1.690(12)	Mo2-O7	1.711(13)	Mo3-O11	1.690(13)
Mo1-O6^{3}	1.970(11)	Mo2-O8	1.705(12)	Mo3-O12	1.707(14)
Mo1-O6	1.970(11)	Mo2-O9	2.207(12)	Cu1-O4^{1}	1.952(11)
Cu1-O2	1.924(12)	Mo2-O10	1.962(11)	Cu1-O6	1.969(12)
Cu1-O9	2.437(12)	Cu1-N3	2.054(15)	O5^{3}-Mo1-O6	96.7(5)
O3^{1}-Mo1-O3^{2}	74.9(6)	O5-Mo1-O3^{2}	90.3(5)	O5-Mo1-O6	99.3(5)
O5^{3}-Mo1-O3^{1}	90.3(5)	O5-Mo1-O3^{1}	165.1(6)	O5-Mo1-O6^{3}	96.7(5)
O5^{3}-Mo1-O3^{2}	165.1(6)	O5^{3}-Mo1-O5	104.5(9)	O5^{3}-Mo1-O6^{3}	99.3(5)
O6-Mo1-O3^{2}	78.9(4)	O6-Mo1-O3^{1}	80.3(4)	O6-Mo2-Mo3	112.8(3)
O6^{3}-Mo1-O3^{2}	80.3(4)	O6^{3}-Mo1-O6	153.7(7)	O6-Mo2-O1	79.2(4)
O6^{3}-Mo1-O3^{1}	78.9(5)	O1-Mo2-Mo3	70.3(3)	O6-Mo2-O9	81.5(5)
O7-Mo2-Mo3	131.4(4)	O8-Mo2-Mo3	103.2(4)	O9-Mo2-Mo3	34.1(3)
O7-Mo2-O1	85.4(5)	O8-Mo2-O1	170.4(5)	O9-Mo2-O1	75.6(4)
O7-Mo2-O6	102.5(5)	O8-Mo2-O6	97.6(5)	O10-Mo2-Mo3	39.0(4)
O7-Mo2-O9	159.6(5)	O8-Mo2-O7	104.2(5)	O10-Mo2-O1	80.3(5)
O7-Mo2-O10	97.1(5)	O8-Mo2-O9	95.0(5)	O10-Mo2-O6	150.2(5)
O9-Mo3-Mo2	42.0(4)	O8-Mo2-O10	99.2(5)	O10-Mo2-O9	72.6(5)
O9-Mo3-O10	77.9(5)	O11-Mo3-Mo2	115.1(5)	O12-Mo3-O10^{4}	93.0(6)
O9-Mo3-O10^{4}	146.9(5)	O11-Mo3-O9	104.2(6)	O2-Cu1-O4^{2}	173.1(5)
O10^{4}-Mo3-Mo2	105.8(3)	O11-Mo3-O10	121.1(6)	O2-Cu1-O6	93.0(5)
O10-Mo3-Mo2	36.4(3)	O11-Mo3-O10^{4}	97.4(6)	O2-Cu1-O9	85.1(5)
O10^{4}-Mo3-O10	69.5(5)	O11-Mo3-O12	107.7(7)	O4^{2}-Cu1-N3	90.8(5)
O2-Cu1-N3	89.2(5)	O12-Mo3-Mo2	129.8(5)	O6-Cu1-O9	75.7(4)
O4^{2}-Cu1-O6	88.7(5)	O12-Mo3-O9	103.7(6)	O6-Cu1-N3	165.5(6)
O4^{2}-Cu1-O9	88.8(4)	O12-Mo3-O10	129.4(6)	Mo2-O6-Cu1	112.4(5)
N3-Cu1-O9	118.8(5)	Mo2-O6-Mo1	124.4(6)	Mo3-O9-Mo2	103.9(5)
C7-O1-Mo2	133.9(12)	Cu1-O6-Mo1	121.9(6)	Mo3-O9-Cu1	147.3(6)
C7-O2-Cu1	127.7(11)	Mo2-O9-Cu1	89.3(4)	C15-N3-Cu1	120.8(13)
C8-O3-Mo1^{1}	132.5(10)	Mo3^{4}-O10-Mo3	110.5(5)	C19-N3-Cu1	122.4(12)
C8-O4-Cu1^{5}	132.0(11)	Mo2-O10-Mo3	104.6(5)		

Mo2-O10-Mo3[4]	144.8(7)				
		Compound **3**			
Co1-O31	1.946(5)	O3-Co12	1.946(5)	O31-Co1-O32	107.5(3)
Co1-O32	1.946(5)	O31-Co1-O2	111.7(2)	O2-Co1-O23	126.6(3)
Co1-O2	1.947(5)	O32-Co1-O2	99.4(2)	O32-Co1-O23	111.7(2)
Co1-O23	1.947(5)	O31-Co1-O23	99.4(2)		
		Compound **4**			
Mo1-O12	1.748(3)	Mo1-Mo2	3.2154(6)	O15-Mo3-O8	100.12(15)
Mo1-O24	1.692(3)	Mo1-O6	1.961(3)	Mo2-O4-Mo4	116.04(15)
Mo1-O26	1.935(3)	Mo1-O10	2.175(3)	Mo1-O6-Mo21	109.33(12)
Mo2-O4	1.895(3)	Mo1-O101	2.361(3)	Mo1-O6-Mo3	109.91(14)
Mo2-O61	2.346(3)	O18-Mo2-O10	160.62(14)	Mo3-O6-Mo21	104.28(11)
Mo2-O10	2.289(3)	O6-Mo1-Mo2	123.29(9)	Mo3-O8-Mo4	117.21(14)
Mo2-O16	1.696(3)	O6-Mo1-O101	77.31(10)	Mo1-O10-Mo11	104.77(12)
Mo2-O18	1.705(3)	O6-Mo1-O10	77.94(11)	Mo1-O10-Mo2	92.13(10)
Mo2-O26	2.023(3)	O101-Mo1-Mo2	86.09(7)	Mo1-O10-Mo3	91.69(10)
Mo3-O6	1.991(3)	O10-Mo1-Mo2	45.35(7)	Mo11-O10-Mo4	92.27(9)
Mo3-O8	1.910(3)	O10-Mo1-O101	75.23(12)	Mo1-O10-Mo4	162.95(13)
Mo3-O10	2.332(3)	O12-Mo1-Mo2	134.16(10)	Mo2-O10-Mo11	98.42(10)
Mo3-O15	1.707(3)	O12-Mo1-O6	96.54(13)	Mo2-O10-Mo3	162.58(13)
Mo3-O19	1.697(3)	O12-Mo1-O101	81.25(11)	Mo2-O10-Mo4	85.45(9)
Mo3-O261	2.337(3)	O12-Mo1-O10	156.47(12)	Mo3-O10-Mo11	97.02(10)
Mo4-O2	1.706(3)	O12-Mo1-O26	97.57(13)	Mo3-O10-Mo4	85.94(9)
Mo4-O4	1.916(3)	O24-Mo1-Mo2	90.76(11)	Mo1-O12-Mo41	117.42(13)
Mo4-O8	1.928(3)	O24-Mo1-O6	100.00(14)	Mo1-O26-Mo2	108.64(13)
Mo4-O9	1.699(3)	O24-Mo1-O101	173.70(13)	Mo1-O26-Mo31	110.43(13)
Mo4-O10	2.471(3)	O24-Mo1-O10	98.70(13)	Mo2-O26-Mo31	103.58(11)
Mo4-O121	2.315(3)	O24-Mo1-O12	104.80(15)	O26-Mo2-Mo1	34.76(8)
O15-Mo3-O10	93.66(13)	O24-Mo1-O26	102.39(14)	O26-Mo2-O61	71.53(10)
O15-Mo3-O261	163.76(13)	O26-Mo1-Mo2	36.59(9)	O26-Mo2-O10	73.92(10)
O19-Mo3-O6	101.27(15)	O26-Mo1-O6	149.42(11)	O6-Mo3-O10	73.67(11)
O19-Mo3-O8	100.93(15)	O26-Mo1-O10	78.31(11)	O6-Mo3-O261	72.27(10)
O19-Mo3-O10	161.16(14)	O26-Mo1-O101	78.22(10)	O8-Mo3-O6	146.82(12)
O19-Mo3-O15	105.06(17)	O4-Mo2-Mo1	121.00(9)	O8-Mo3-O10	77.36(11)
O19-Mo3-O261	89.60(14)	O4-Mo2-O61	83.87(12)	O8-Mo3-O261	83.54(11)
O2-Mo4-O4	99.25(15)	O4-Mo2-O10	78.49(11)	O9-Mo4-O121	91.06(14)
O2-Mo4-O8	99.10(15)	O4-Mo2-O26	147.51(12)	O121-Mo4-O10	69.04(9)
O2-Mo4-O10	94.22(13)	O61-Mo2-Mo1	78.94(7)	O10-Mo3-O261	71.56(9)
O2-Mo4-O121	163.26(14)	O10-Mo2-Mo1	42.52(7)	O15-Mo3-O6	97.56(14)
O4-Mo4-O8	143.33(13)	O10-Mo2-O61	71.77(9)	O18-Mo2-O61	89.03(13)
O4-Mo4-O10	73.58(11)	O16-Mo2-Mo1	84.49(11)	O8-Mo4-O10	73.64(11)
O4-Mo4-O121	76.35(12)	O16-Mo2-O4	102.49(15)	O16-Mo2-O61	163.20(13)
O8-Mo4-O121	76.96(12)	O16-Mo2-O10	94.04(12)	O18-Mo2-O26	97.95(14)
O9-Mo4-O2	105.68(17)	O16-Mo2-O18	104.45(16)	O18-Mo2-O4	102.63(15)

O9-Mo4-O4	103.22(15)	O16-Mo2-O26	96.29(14)	O9-Mo4-O10	160.09(14)
O9-Mo4-O8	101.96(15)	O18-Mo2-Mo1	132.62(12)		
